# A Combined Experimental and Modeling Study for Pellet-Fed Extrusion-Based Additive Manufacturing to Evaluate the Impact of the Melting Efficiency

**DOI:** 10.3390/ma14195566

**Published:** 2021-09-25

**Authors:** Andrea La Gala, Rudinei Fiorio, Daniel V. A. Ceretti, Mustafa Erkoç, Ludwig Cardon, Dagmar R. D’hooge

**Affiliations:** 1 Centre for Polymer and Material Technologies (CPMT), Department of Materials, Textiles and Chemical Engineering, Ghent University, Technologiepark, 130, Zwijnaarde 9052, 9000 Ghent, Belgium; Andrea.LaGala@UGent.be (A.L.G.); rudinei.fiorio@UGent.be (R.F.); daniel.ceretti@ugent.be (D.V.A.C.); mustafa.erkoc@ugent.be (M.E.); ludwig.cardon@ugent.be (L.C.); 2 Centre for Textiles Science and Engineering (CTSE), Department of Materials, Textiles and Chemical Engineering, Ghent University, Technologiepark, 70A, Zwijnaarde 9052, 9000 Ghent, Belgium; 3 Laboratory for Chemical Technology (LCT), Department of Materials, Textiles and Chemical Engineering, Ghent University, Technologiepark, 125, Zwijnaarde 9052, 9000 Ghent, Belgium

**Keywords:** rapid prototyping, 3D printing, melting single screw extrusion, material design

## Abstract

To improve the product quality of polymeric parts realized through extrusion-based additive manufacturing (EAM) utilizing pellets, a good control of the melting is required. In the present work, we demonstrate the strength of a previously developed melt removal using a drag framework to support such improvement. This model, downscaled from conventional extrusion, is successfully validated for pellet-based EAM—hence, micro-extrusion—employing three material types with different measured rheological behavior, i.e., acrylonitrile-butadiene-styrene (ABS), polylactic acid (PLA) and styrene-ethylene-butylene-styrene polymer (SEBS). The model’s validation is made possible by conducting for the first time dedicated EAM screw-freezing experiments combined with appropriate image/data analysis and inputting rheological data. It is showcased that the (overall) processing temperature is crucial to enable similar melting efficiencies. The melting mechanism can vary with the material type. For ABS, an initially large contribution of viscous heat dissipation is observed, while for PLA and SEBS thermal conduction is always more relevant. It is highlighted based on scanning electron microscopy (SEM) analysis that upon properly tuning the finalization of the melting point within the envisaged melting zone, better final material properties are achieved. The model can be further used to find an optimal balance between processing time (e.g., by variation of the screw frequency) and material product performance (e.g., strength of the printed polymeric part).

## 1. Introduction

Over the past few years, the use of additive manufacturing (AM) has significantly increased in the production of polymeric products and material shapes [1,2,3,4,5,6,7,8,9,10]. Most polymeric AM applications start with a filament [9,10,11,12], which is forced through a heated nozzle, promoting its melting to then deposit consecutive material layers. This is performed according to geometric and process parameters that can have a strong influence on the properties of the final parts [13,14,15,16]. In recent years, however, a growing number of AM applications introduced the use of single-screw extruders (SSEs) fed with polymer pellets [17,18,19,20,21]. The global term for such AM modifications is extrusion-based AM (EAM), complementary with conventional large-scale extrusion [22,23,24,25,26].

EAM allows us to overcome several limitations of filament-based AM, which is also known as fused filament fabrication (FFF). Specifically, soft polymers can be manufactured that are difficult to process through traditional 3D printing [19,20]. Printing employing polymer pellets leads to a lower cost of the final material [20] and, due to the higher possible melting rate [19], a faster processing speed for, in principle, a wider range of materials is realized [20]. It allows us to bypass the filament extrusion process that normally precedes the AM process, relieving the material of one extrusion cycle, leading to less degradation [27].

One of the remaining EAM challenges is to select, for a given polymeric material, the appropriate operating conditions to avoid/minimize both thermal degradation and solid slippage. Here, control of the melting zone is crucial. However, this is far from trivial, as the melting process depends on many interdependent parameters, ranging from process parameters such as the barrel temperature and screw frequency to the shear dependent viscosity and stress, and eventually to material properties such as density, thermal conductivity, and heat capacity [28,29,30,31,32,33,34,35,36,37]. Unfortunately, only a limited number of studies have investigated EAM melting, as (i) typical AM research is devoted to the macroscopic analysis of the printed product as such and (ii) EAM has only emerged as a key production technique this century. If melting is investigated, one needs to refer to studies on large scale extrusion, in which the dimensions are of a totally different order of magnitude [28,29,30,31,32,33,34,35,36,37].

Looking back at the long history of conventional extrusion, Maddock [28] was the first to experimentally investigate the melting mechanism in conventional SSE. Later on, Tadmor developed [29] and experimentally validated [30,31] an analytical model by considering a heat and mass balance for the melt film between the solid bed and the barrel. This model was then modified to include the effects of shear rate and temperature on the viscosity [24]. Upon accounting for shear thinning with, for instance, a power law index and assuming melt removal by drag, the number of screw rotations or, equivalently, the unwounded screw length at which melting stops for a given set of processing conditions could be more reliably predicted.

In parallel with the work and modifications of Tadmor, other large-scale extrusion melting models have been developed and applied. Donovan [32], for example, focused on the melting efficiency, assuming a constant melt film thickness and an identical down-channel velocity for both the solid bed and the melt pool at any cross section. This model allowed, at first glance, a good prediction of the melting behavior, but was later criticized for the poor prediction of the overall melting profiles [33]. Edmondson and Fenner [34] introduced a model that accounts for the presence of a molten film between the screw and the solid bed, which is thus an extra drag contribution compared to the bed and barrel. The predictions of the model appeared to be satisfying for both the solid bed and pressure profiles. Furthermore, Mount et al. [35] proposed and satisfyingly validated analytical equations for the melting rate prediction that did not require an iterative calculation. This was done by reducing part of the constitutive equations to non-dimensional forms, employing characteristic scales and non-dimensional numbers.

Syrjala [36] in turn evaluated the melting profile for SSEs by only using relevant flow field equations, without any focus on the mechanism of melting. Notably, in a more recent work, Altınkaynak [37] performed three-dimensional finite element simulations of the melting process by solving the conservation equation for mass, momentum, and energy along with a generalized Newtonian constitutive equation. Such model efforts thus go beyond analytical approximate solutions, but the solving of the related equations is too slow in the context of online process control.

In our recent work [38], we theoretically studied the efficiency of EAM melting by downsizing Tadmor’s pioneering melting mechanism from conventional large-scale extrusion to micro-extrusion, selecting acrylonitrile–butadiene–styrene (ABS) as a viscoelastic reference polymer. We highlighted that the variation of the Brinkman (Br) number [39,40,41,42,43], which is the ratio between the melting contribution through heat dissipation and that through conduction, along the unwounded screw length is different for EAM compared to conventional SSE. This Br difference is due to a different balance of shear rates, melt layer thicknesses and screw geometry, consistent with speculations in previous studies [44,45,46]. In the process of scaling down SSE, a reduction in the viscous dissipation was thus witnessed in our previous work. This could be made clearer upon comparing the melting profiles for micro-extrusion and conventional extrusion under typical operation conditions for each technique on its own [38], consistent with preliminary results [47]. We also put forward in our previous theoretical study that screw parameters such as the screw frequency and pitch angle and material parameters such as the power-law index influence the relative position of the final EAM melting point.

One can thus not generalize the melting behavior of one polymer directly to another, highlighting the relevance of the development of more generic theoretical frameworks to support the identification of ideal EAM conditions. Furthermore, it can be expected that good printed macroscopic properties require a good melting. Consistent with this, the relevance of an adequate melting was also presented in previous studies for PLA [48] and ABS [49], showing how low printing temperatures can cause a steep decrement in the properties of the final parts. Hence, an understanding of the melting can help to better identify better printing conditions.

In the present work, we therefore further increase the impact of our modeling framework by benchmarking the EAM melt model to in-house determined experimental data. To record these data, we perform a series of so-called screw-freeze experiments [28] to visually inspect the positioning of the solid bed and melt pool at distinct screw positions. The material portfolio is also expanded beyond simply ABS to include several rheological behaviors. The first polymeric material ABS is characterized by an amorphous structure, the second polymeric material is polylactic acid (PLA) which has, in contrast, a semicrystalline structure, and the third polymeric material is styrene-ethylene-butylene-styrene polymer (SEBS), which falls into the class of thermoplastic elastomers. A comparison is included regarding the melting mechanism for the three different materials, through a comparison of the Brinkman number variation [39,40,41,42,43]. Moreover, the quality of the printed parts is related to the melting efficiency, as accessible for any set of operating conditions through application of the modeling framework. In addition, a first step toward a full macroscopic property analysis is included by including imaging and tensile results.

## 2. Materials and Micro-Extruder Machine Details

In this study, three commercially available extrudable polymeric materials were used: (i) acrylonitrile butadiene styrene polymer (ABS) MagnumTM M3404 from Dow Chemical Company (Midland, MI, USA), (ii) polylactic acid (PLA) Ingeo ™ 3D850 from NatureWorks (Minnetonka, MN, USA), and (iii) styrene-ethylene-butylene-styrene polymer (SEBS) KRATON^TM^ G1657M from Kraton (Huston, TX, USA). Table 1 summarizes the most important materials characteristics. Additionally, a small amount of Clariant Plastiflac polyethylene (PE)-based red pigment was used for the screw-freezing experiments.

The micro-extrusion machine geometry is the same for all of the material processing and simulations, and its details are reported in Table 2. The dimensions of the geometry are consistent with our previous work [38] and resemble a typical micro-extruder design.

## 3. Experimental and Theoretical Methods

### 3.1. Melting Model

The melting profile was simulated using the melting model and the micro-extruder geometry described in our previous theoretical study [38]. For completeness, the main model parameters are reported in Table 3, making a differentiation between the three polymer types and extracting values from the previous Tables (Table 1, Table 2, Table 3, Table 4, Table 5 and Table 6). Essential in the model is the calculation of the melting rate per unit of channel width *q*:

(1) $$q=\sqrt{\frac{X\;\rho_{m}\;\;|V_{\mathrm{bx}}|}{\;\lambda +c_{s}\;\left(T_{m}-T_{0}\right)+c_{m}\;\Theta \;\left(T_{b}-T_{m}\right)}\left(T_{b}-T_{m}\right)k\left(1+\frac{B_{r}}{2}\right)}$$

In this equation, reported from our previous work [38], *X* is the width of the solid bed (initially *X* is equal to the channel width *W*), $\;\rho_{m}\;$ is the density of the molten phase, *V*_bx_ is the velocity of the molten phase at the barrel in the *x* direction (as reported in Figure S1 of the Supplementary Materials), λ is the heat of fusion, *c*_m_ is the specific heat capacity of the molten phase, *c*_s_ is the specific heat capacity of the solid phase, *Θ* is the average temperature of the melt that is related to the Brinkman number Br [39,40,41,42,43], *T*_m_ is the melting temperature, *k* is the thermal conductivity, and *T*_b_ is the barrel temperature.

Br is a dimensionless number that indicates the ratio between the viscous heating and the (molecular) conduction heating [39,40,41,42,43]. The viscous heating is generated by the friction between the flowing elements at different velocities and is finally supplied by the engine that turns the extrusion screw. The conduction heat, on the other hand, is supplied by the external heater bands. Br is defined in Equation (2), reported from our previous work [38], and was used to evaluate differences in the melting mechanism between the materials tested.

(2) $$\mathrm{Br}=\frac{\eta \;V_{j}^{n+1}}{{\bar{\delta }}^{n-1}\;k\left(T_{b}-T_{m}\right)\;}$$

In Equation (2), *η* is the shear rate dependent melt viscosity, *V*_j_ is the velocity difference between the velocity of the solid bed in the *z* direction *V*_sz_ and the velocity of the melt layer at the barrel surface *V*_b_, *n* is the pseudo-plasticity index, and $\bar{\delta }$ is the average thickness of the molten layer that can be assessed and updated along the unwounded screw as explained in our previous work [38]. Note that *V*_bx_, reported from our previous work [38], is a function of the screw frequency *N*, the inside diameter of the extruder barrel *D*_b_, and the helix pitch angle of the screw Ɵ:

(3) $$V_{\mathrm{bx}}=V_{b}\;sinƟ=\pi \;D_{b}\;N$$

It should be stressed that *V*_sz_ is dependent on the volumetric flow rate at the inlet of the extruder, *Q*_0_. The value of *Q*_0_ was estimated by assuming stationary conditions in the extruder and weighing the extruded amount for defined intervals of time. Hence, no ball-park value was selected, but for each polymeric material, a true value based on actual steady-state experiments was considered.

### 3.2. Rheological Measurements and Cross Model Fitting

Small amplitude oscillatory shear (SAOS) tests were performed using a MCR 702 rheometer (Anton Paar, Graz, Austria). The storage modulus (G′) and loss modulus (G′′) were monitored as a function of frequency (1 × 10^−1^ to 6 × 10^2^ rad s^−1^), using the parallel plate configuration with a 25 mm diameter, a gap of 1 mm and an amplitude of 1%. The tests were performed under nitrogen atmosphere at the same (target or average) processing temperatures used for the actual EAM experiments utilizing the operating settings as reported in Table 3. The latter table provides an overall summary of parameters directly relevant for the results and discussion. Specifically for ABS, the processing temperature is higher, as preliminary screening has shown that the viscosity is higher. The disks were obtained by compression molding of dried pellets at the same temperatures, considering a diameter of 25 mm and 1 mm thickness. The discs were also dried before the rheological measurements.

**Figure 1:**
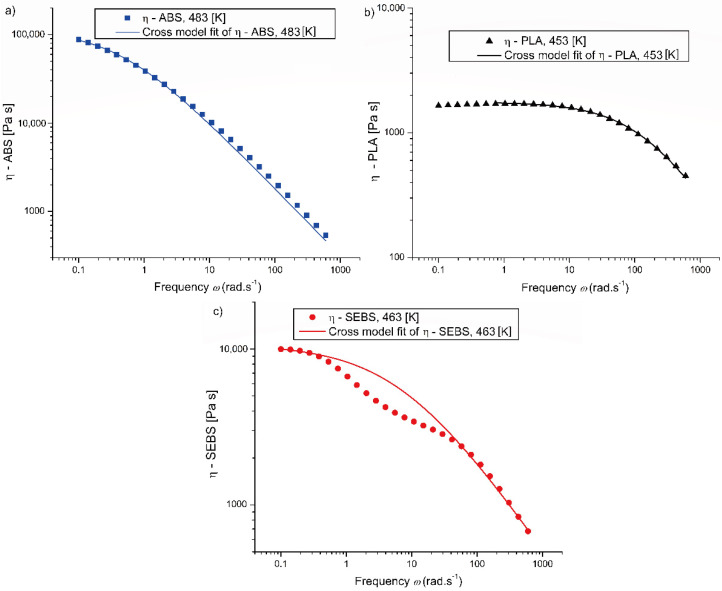
Viscosity as a function of the frequency for (**a**) ABS, (**b**) PLA, (**c**) and SEBS (specifications in Table 1), taking the same temperatures as the (target/average) EAM processing parameters. Fitting parameters via the Cross model listed in Table 3.

As shown by the symbols in Figure 1, the three polymeric materials, with specifications in Table 1, globally have a similar rheological behavior, with the viscosity *η* exhibiting a shear thinning behavior after the Newtonian region, as is most evident in the case of PLA, shown in Figure 1b. There are, however, keeping in mind the different temperatures at which the curves were obtained, some clear differences in the values for (*η*_0_), which is the Newtonian viscosity limit before the critical shear rate (*γ*_cr_). It particularly follows that ABS is considerably more viscous than SEBS, with PLA exhibiting the lowest (*η*_0_) value among the three polymeric materials. A previous study [58] observed that the rheological behavior of ABS is strongly dependent on the (average) molar mass of the SAN (styrene-acrylonitrile) phase. In particular, an increase in the mass average molar mass of the SAN phase leads to the increase in viscosities under both shear and uniaxial extension. This increment in average molar mass leads to longer SAN chains being grafted onto the PB (poly-butadiene) particles. This results in the formation of a denser structural “network”, with a higher yield stress and storage modulus in the low frequency plateau region. For completeness, it is mentioned here that strong shear thinning effects are also indicative of a wide molar mass distribution for the SAN phase [59]. Furthermore, for SEBS, a more complex rheological behavior is recorded in Figure 1c. In a previous work [60], it has been observed that the shear thinning effect in the region between 0.1 and 1.0 rad s^−1^ is only present at temperatures at which the SEBS’ microphase-separated state changes into a single-phase state, which is called the order-disorder transition (ODT). This effect is a strong indication of the presence of several melt structures, and the extreme shear thinning behavior at low shear rates can be associated with a melt yield stress.

The experimental rheological data for each polymer were also used to formally fit a Cross flow model at the selected temperature:

(4) $$\eta =\frac{\hat{\eta_{0}}}{1+{\left(\frac{\eta_{0}\;\dot{\gamma }}{\tau^{*}}\right)}^{1-n}}$$

(5) $$\hat{\eta_{0}}=\eta_{0}(1+{\left(\frac{\eta_{0}\;\dot{\gamma_{\mathrm{cr}}\;}}{\tau^{*}}\right)}^{1-n})$$

In Equations (4) and (5), reported from our previous work [38], *η* is the shear rate dependent melt viscosity, *n* is the pseudo-plasticity index (or shear thinning factor), τ* is the critical Cross model stress at the transition to shear thinning, and *γ* is the shear rate (formally assumed equal to the frequency), while $\hat{\eta_{0}}$ is a short notation to retrieve the Newtonian limit thus before the critical shear rate $\dot{\gamma_{\mathrm{cr}}\;}$.

The corresponding fits are highlighted as lines in Figure 1a–c, and the Cross-model parameters are reported in Table 3. It follows from Figure 1 that for ABS and PLA, good descriptions are obtained with the Cross model, but for SEBS, the curve’s fit is less accurate. A cross-model only grasps an average overall behavior or one structure, and thus not the ODT.

### 3.3. Thermal Properties

The thermal conductivity *k* of the polymeric samples was measured using the Transient Plane Source method with a Hot Disk TPS 2500S (Hot Disk, Göteborg, Sweden) according to ISO 22007-2. The conditions of the experiments are reported in Table 4. The *k* value was obtained by an average of 3 measurements and is assumed to be representative (during the processing) at all temperatures by taking the average over all data in Table 4.

The specific heat capacities *c*_s_ (solid) and *c*_m_ (melt), the heat of fusion λ, and the “melting” temperature *T*_m_ of the polymeric materials were evaluated through differential scanning calorimetry (DSC), using a Polyma DSC 214 from Netzsch. The measurements were obtained using the ASTM standard E1269, which requires three scans: (i) a baseline scan, (ii) a scan using a sapphire standard, and (iii) the sample scan. The specific heat capacity was measured at 300 K, which was the initial temperature of the three materials, and at 460 K, at which all materials were in the molten state. The experiments were conducted at a heating rate of 5 K min^−1^. The conditions and results are reported in Table 5. Again, constant (average) values were considered in what follows, further supported by the sensitivity analysis in our previous work [38].

To take into account a possible difference in the PLA crystallinity (special case in Table 1 regarding crystallinity) between the injection molded samples for the Hot Disk and the solid pellets for EAM, further DSC tests were conducted at 10 K min^−1^. A variation of only 1.70% was found for the area of the crystalline peak, as shown in Figure S2 in the Supporting Information. The effect of this variation in the PLA crystallinity during the sample preparation was neglected, as previous studies observed a marginal impact of the degree of crystallinity on the thermal conductivity [61]. Moreover, previous studies on the melting mechanisms in SSEs observed that small variations in the thermal conductivity have a neglectable impact on the solid bed profile (SBP) [21,38].

### 3.4. Solid and Melt Density

The density of the solid phase was measured according to the Immersion Method of ISO/DIS 1183-1, weighing the pellets in air and in ethanol as an auxiliary liquid. The weight scale used was a Precisa XR205SM-DR manufactured by Precisa Gravimetrics AG (Dietikon, Switzerland).

A Melt Indexer MPX 62.92 by GÖTTFERT ^®^ (Buchen, Germany) was used to measure the Melt Volume Rate (MVR) of the different polymeric materials under the conditions described in Table 6, according to ISO 1133:2005 and ISO 1133:2005 Cor.1:2006. Under the same conditions, the mass flow rate (MFR) was estimated by weighing the output from the melt indexer for a given time period. The melt density was evaluated as the ratio between MFR and MVR. The results are also presented in Table 6. The final density values were then averaged and used at any temperature for the processing simulations.

### 3.5. Screw-Freezing Experiments

**Figure 2:**
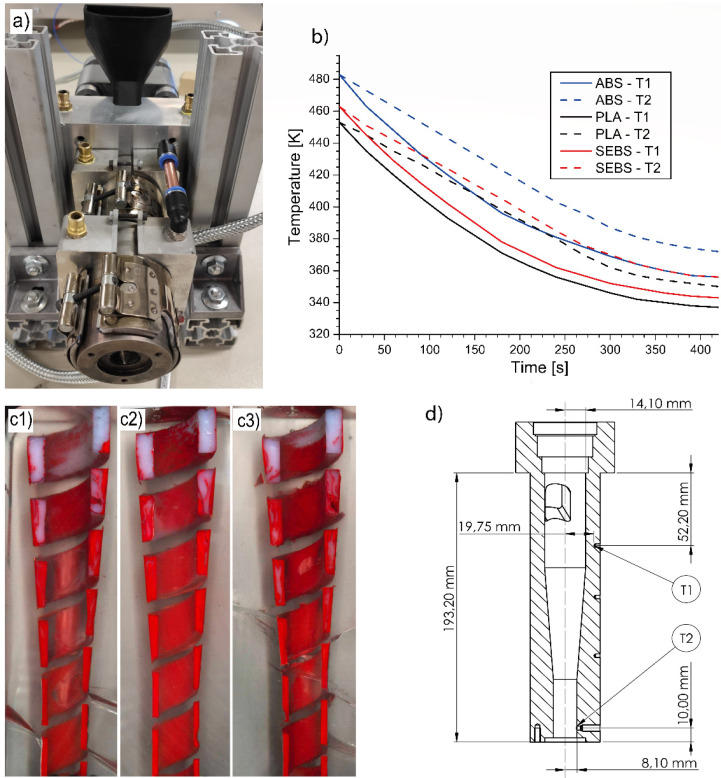
(**a**) Micro-extruder cooling setup for screw-freezing experiments and (**b**) micro-extruder barrel cooling chart after stopping the extrusion with active water-cooling (T_1_: thermocouple 1), (**c1**–**c3**) embedded samples for ABS at screw frequency of 2, 5, and 8 rpm, respectively, from left to right, (**d**) position of the thermocouples, units in mm.

The EAM melting model predictions were validated through a series of screw-freezing experiments that were conducted through rapid water cooling of the operating micro-extruder (Ghent University, Gent, Belgium). This was performed through water-cooled aluminum bands that were positioned along the extruder barrel, as visible in Figure 2a. After a screw-freezing experiment was completed, the extruder was disassembled. Then, the solidified polymer channel was extracted from the barrel and cut into sections to validate the model predictions on SBP.

The screw freezing technique was first introduced by Maddock for conventional extruders [23]. This technique starts with the extruder running in steady state. The screw rotation is then stopped, and the barrel is air cooled to solidify the polymer in the screw channel. The screw with the solidified polymer is extracted and the cross-sections of the solidified polymer are polished and investigated. In the present work on micro-extruders, the same approach was applied, although some modifications were made. In greater detail to highlight the regions where the polymer was molten, a small amount of PE pigmented feedstock polymer was introduced at a mass ratio of 0.5:100. The solid bed still showed the natural color of the pellets, while the regions where the polymer had melted were strongly colored by the pigment, according to the same principle described in Maddock’s study [28]. Additional water-cooling rings were added to the compression and metering sections of the extruder barrel to improve the cooling behavior. The cooling rings were assembled to promote the cooling of the lower section first and then the upper section. The cooling curves for the two sections after EAM competition are depicted in Figure 2b, with the positions of the thermocouples visible in Figure 2d. It follows that a relatively efficient cooling is realized.

For most of the tests with ABS, the solidified polymer channel had a negligible deformation during the extraction process, which allowed for epoxy embedding to better showcase the melting mechanism and the SBP. The embedded samples were finally polished, and a high-resolution scanner was used for the image acquisition, with the results depicted in Figure 2 for three selected screw frequencies. For the tests with PLA, SEBS, and two specific ABS tests, due to the deformations or fractures of the channel during the extraction process, it was impossible to replicate the methodology used for the previous samples mentioned. After cutting and polishing of the sections, the image was acquired through a camera. Examples of sections for the non-embedded samples are visible in Figure S3 of the Supporting Information.

**Figure 3:**
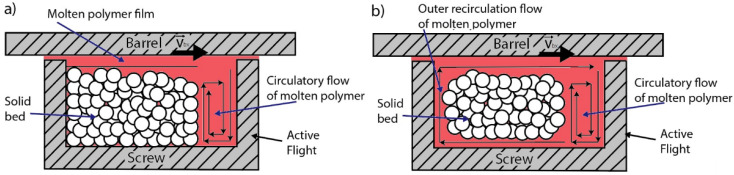
Melt removal by the drag mechanism: (**a**) reported by Maddock and Tadmor [28,29,30,31] for large scale SSEs and initially observed in the present work; (**b**) outer recirculation flow observed in several cases in the conical screw micro-extruder. In the end, the overall measuring/modeling result remains the same for subplot a and b through quantification of the area of the molten part vs. the total available space.

Similarly to what was observed by Maddock and Tadmor [28,29,30,31] for conventional extrusion, the polymer melt appears at first sight as a film in between the hot barrel and the compacted pellets, as conceptually shown in Figure 3a. Advancing along the channel, however, the recorded behavior departs from what Maddock and Tadmor observed [28,29,30,31], due to the clear presence of an outer recirculation zone, as conceptually depicted in Figure 3b. This can be explained by the different geometry of the micro-extruder, in particular the conical shape of the screw and barrel that leads to a substantial variation in the velocity profiles, as pointed out in our previous work [38].

Due to the presence of such outer recirculating flow, the width of the molten phase was not constant along the solid bed (in a given screw element), and the percentage of unmolten area over the total area was selected to represent the ratio of the solid bed width and screw width for comparison with the numerical model predictions. The area (A) analysis was executed using the IMAGEJ image processing and analysis software (Version 1.8.0, U. S. National Institutes of Health, Bethesda, MD, USA) for the different sections. For the non-embedded samples, a different image acquisition interpretation was found to be related to the separation of the two phases, due to the lack of a very clear separation in the color distributions in contrast to the very clear color contrast for the embedded samples. The threshold for these cases was selected based on visual feedback, in coherence with the melting mechanism. Too high or low values of the threshold would, for example, show the presence of molten or solid particles in positions not consistent with the expected melting mechanism.

### 3.6. SEM Analysis of Extruded and Solidified Samples

A Phenom Pro X Desktop Scanning Electron Microscope (SEM) from ThermoFisher Scientific (Waltham, MA, USA) was used to investigate the surfaces of extruded filament samples at different screw frequencies. The images were acquired using a backscatter electron detector (BSD) technique with a beam energy of 15 keV with a magnification of 490:1.

The scope of the analysis was to investigate the presence of surface defects caused by prolonged residence times, or by the presence of solid particles in the metering section. The selected frequencies were 0.5, 5 and 20 rpm, based on model screening, allowing us to identify the position of the point of melt finalization. Two filament samples were always collected from the extrusion under steady state conditions, at a 15 min interval. The process was repeated using 2 nozzles with 0.8 and 1.5 mm final opening diameter, for a total of 12 samples.

### 3.7. Tensile Testing of the Extruded Filaments

An Instron 5566 tensile machine (Instron, Norwood, MA, USA) was used to investigate the tensile properties of filaments of ABS extruded at different screw frequencies. The method used for the tensile test was based on a previous work [62]. A constant velocity of 10 mm·min^−1^ was used to evaluate the stress–strain relationship. The gauge length was set at 50 mm. Again, the selected screw frequencies were 0.5, 5 and 20 rpm.

## 4. Results and Discussion

### 4.1. Model Validation Regarding the Point of Melt Finalization for Three Polymeric Materials

**Figure 4:**
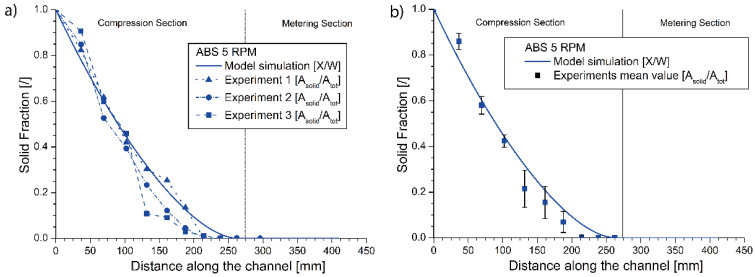
(**a**) Predicted and experimental individual melting profiles for ABS for three runs at 5 rpm; (**b**) predicted profile and average experimental points for ABS processed at 5 rpm (Table 3).

Three screw-freezing experiments were first performed for the ABS polymer at a nominal screw speed of 5 rpm, and the results after data analysis are reported in Figure 4a (symbols with connections). The average (mean) values of these experimental results are compared in Figure 4b with the model predictions, inputting the rheological data from Figure 1a (parameters in Table 3). For completeness, these modeled data are also reported in Figure 4a. It follows from Figure 4b that the melting is performed in the compression section at ca. 250 mm and the decrease in the solid fraction is gradual and sufficiently captured by the model. The details of the image processing steps are provided in Figures S4–S6 of the Supplementary Materials, with, in the case of the first 2 Supplementary Materials figures related to experiment 1 and 2, non-embedded samples being considered, and for the last figure (Figure S6 of the Supplementary Materials), focus is on the embedded sample (experiment 3).

**Figure 5:**
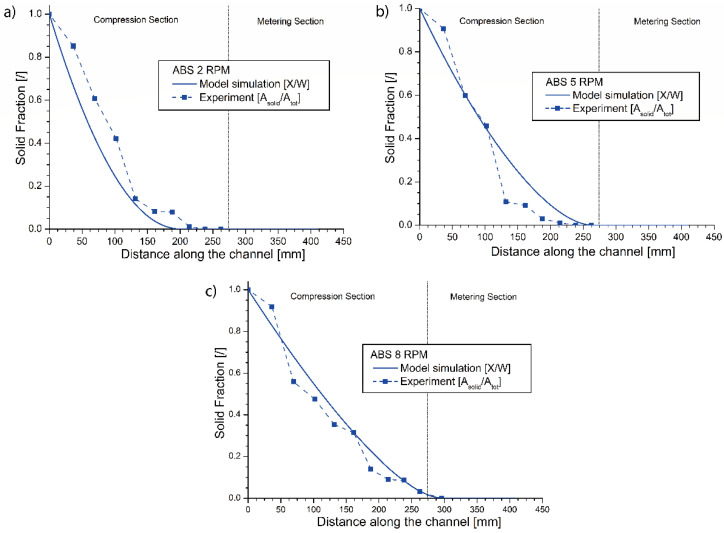
(**a**) Predicted and experimental melting profiles for embedded ABS samples at: (**a**) 2 rpm; (**b**) 5 rpm; (**c**) 8 rpm (Table 3).

For ABS, two additional experiments were performed at 2 and 8 rpm to evaluate the effect of an increment and a decrement in the screw speed. The results are depicted in Figure 5a,c directly as average experimental results, also including the model predictions. For the sake of comparison, the ABS results from before at 5 rpm are also repeated in Figure 5b. The details of the image processing are visible in Figure S7 of the Supplementary Materials for the 2 rpm test and in Figure S8 of the Supplementary Materials for the 8 rpm test. In Figure 5, a good agreement is observed between the experimental results and the numerical model prediction for all three screw frequencies, also realizing that a model can wipe out experimental issues, in the present work due to non-embedding. Closer inspection reveals that the observed final differences between the 2 and 5 rpm results are still safely within the limits of the envisaged melting zone (but with 50 mm difference), while for 8 rpm, a shift in the melting profile to higher values is observed, even up to 300 mm, which is outside the envisaged melting zone.

A further interpretation of Figure 5a–c also enables a better assessment of the experimental errors and the relevance of the melting model. For example, for 2 rpm, the observed difference between the position of the point of melt finalization in the numerical model and the experiments is around 17.9% for the ninth experimental point. Focusing on the eighth experimental point at which the image processing delivers an estimate of 98.89% of molten material, the difference drops to about 8.2%. For 5 rpm, in turn, the observed difference is around 9.2% for the 9th experimental point. Considering the eighth experimental point, at which the molten fraction is estimated as 97.5%, the difference now increases to 18.8%. For 8 rpm, the observed variation is around 1.5% for the 10th experimental point, with the next point exhibiting about 96.4% of solid material and a 0.8% distance from the experimental profile. Hence, one can indeed state that the model filters out natural experimental errors and is a more reliable tool to fully extrapolate the final point of melting.

As shown in Figure S9a of the Supplementary Materials, the accuracy of the simulations’ results depends on the correctness of the input parameters. Employing the *Q*_0_ values from Table 3, the modeling outcome is in good agreement with the experimental results (full lines). Instead, the use of ballpark *Q*_0_ values, as considered in previous theoretical work [38], is too approximate (dashed lines). The need of benchmarked input parameters is further highlighted in Figure S11 of the Supplementary Materials, showing that for the ABS case, the use of the PLA and SEBS *Q*_0_ and n values from Table 3 are incorrect. Furthermore, in a previous study on SSEs [63], Mount and Chung also highlighted the importance of the method used to evaluate the solid bed velocity.

**Figure 6:**
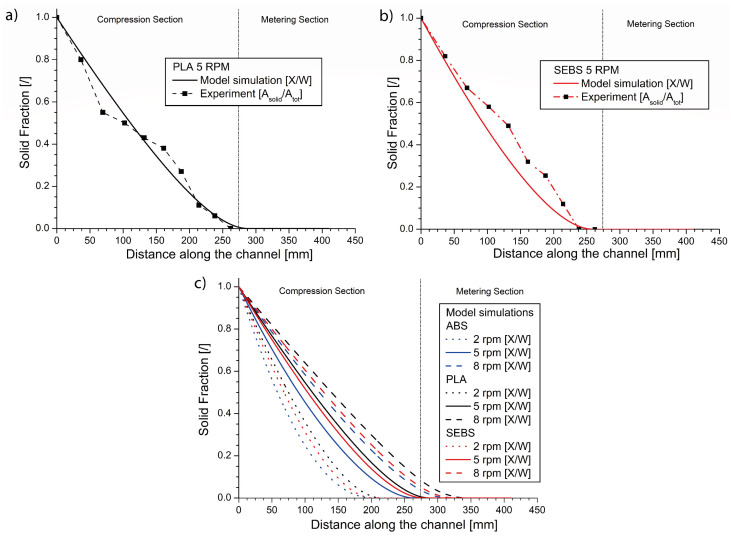
(**a**) Predicted and experimental melting profiles for PLA; (**b**) predicted and experimental melting profiles for SEBS; (**c**) predicted melting profiles for ABS, PLA, SEBS for different screw frequencies (Table 3).

In view of extra model validation, a single screw freezing experiment has been additionally performed for the PLA and SEBS polymeric materials at the nominal screw speed of 5 rpm. The experimental (symbols) and modeling (lines) data are depicted in Figure 6a,b, again displaying the average experimental results. The corresponding results from the image processing are now visible in Figures S9 and S10 of the Supplementary Materials. It is worthwhile mentioning that while the color distribution for ABS and PLA clearly shows in most cases a bimodal distribution, allowing for a clear separation between the different phases, for SEBS, the threshold for separation between the two phases could only be obtained based on visual feedback. As shown in the Supplementary Materials, the SEBS distribution is not clearly bimodal, and thus this somewhat more arbitrary approach for melt efficiency determination is the most recommended.

It follows from Figure 6a,b that the numerical model predictions are again in good agreement with the experimental data, showing a good overall reliability, taking into account the different morphology of the materials in Table 1 and a wide range of viscosities, as expressed in Figure 1 and Table 3. For PLA, a difference in the position of the experimental and simulated point of melt finalization of about 12.3% is observed, while for SEBS, this difference is about 6.9%. Hence, the model is capable of tackling different material morphologies, highlighting the model’s efficacy in preventing experimental errors. Closer inspection shows that the melting for PLA is slightly slower and takes place for longer than the envisaged melting zone, while SEBS melting is still within the envisaged melting zone and is performed at a similar position as for ABS (Figure 5b). Overall, it can thus be concluded that the processing temperature variation in Table 3 allowed us to create similar global behavior in melting. Hence, this operating condition is crucial in the overall design.

A detailed model-driven approach is thus within reach if more rheological data and Arrhenius parameters regarding rheology are available, facilitating automated model screening. In this context, the potential of the model is further highlighted in Figure 6c by comparing at different rpm the melting behavior for the three polymeric materials. It follows that the relative trend (melting faster for the order PLA, SEBS and ABS) is always respected but a different rpm threshold can exist for each material type to obey the melting zone idea of the original micro-extruder design.

### 4.2. The Impact of the Polymer Material Type on the Melting Mechanism

**Figure 7:**
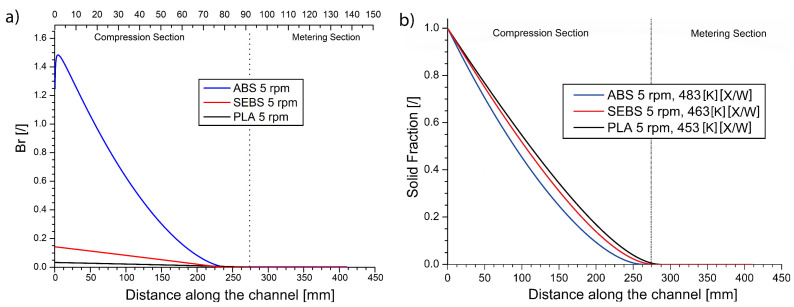
Predicted Brinkman number (**a**) and predicted melting profiles (**b**) for ABS (blue), SEBS (red) and PLA (black) at 5 rpm. The lines in (**b**) are taken from Figure 5 and Figure 6 (Table 3).

Combining the modeled data in Figure 5 and Figure 6, the melting behavior can also be studied at a fixed rpm, varying the material type. This is conducted in the present subsection, specifically at 5 rpm. While the position of the point of melting finalization at 5 rpm moves, e.g., by 8% between ABS and PLA, as shown in Figure 7b, the underlying melting mechanism, as included in Figure 7a, shows a very different behavior. In this subplot, focus is on the variation of Br, with lower values of this number indicative of a higher share of the thermal energy being supplied by the external heater bands compared to the viscous heat generated by friction.

It follows from Figure 7a that the Br values show a very large difference in the heating mechanism for the different materials. While for the ABS material the viscous heat generated is initially still higher than the conduction heat (Br > 1), for the SEBS material, the viscous heat always represents a small fraction, and for the PLA material, it appears to be negligible. The reason for the difference in the melting mechanism can be explained by the differences in the viscosity profiles highlighted in Figure 1, and again highlights the relevance of the rheological curves.

### 4.3. Impact of Melting Efficiency on 3D Printed Properties

**Figure 8:**
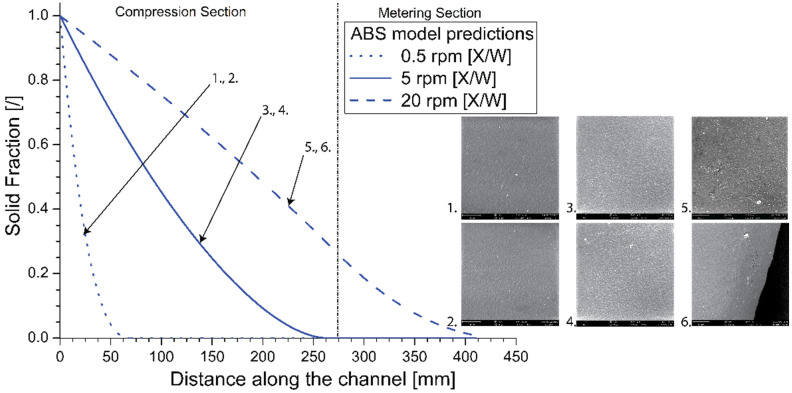
Left: Model predictions for melting profiles for ABS at screw frequencies of 0.5, 5, 20 rpm. Right: SEM images of printed filaments; enlarged and extra SEM images are included in Supplementary Materials Figures S12–S17.

To evaluate the impact that bad melting can have on the final 3D printing applications, the surface of the extruded filament, selecting first a nozzle diameter of 0.8 mm, was analyzed with SEM. For a meaningful comparison, sufficiently different frequencies were selected so that the point of melt finalization is located early in the compression section (0.5 rpm case), at the end of the compression section (5 rpm case; reference case from before), and at the end of the metering section (20 rpm case). This is further confirmed by the model lines in Figure 8. For the 20 rpm case, in particular, the solid bed at the end of the metering section still accounts for about 0.9% of the section area. The SEM images, always recorded based on data in replicate format, are presented as subplots with the arrows highlighting the rpm value.

While the SEM images for both a screw frequency of 0.5 and 5 rpm show a similar surface roughness, the surface for the filament extruded at 20 rpm presents a higher number of defects and a higher roughness. As shown in Figures S15–S17 in the Supplementary Materials, the same differences in roughness are observed in case the nozzle diameter is equal to 1.5 mm instead of 0.8 mm. It is worthwhile mentioning that no substantial variation in *Q*_0_ was observed after the modification of the nozzle diameter. Hence, limitations exist regarding the melting efficiency to enable good material properties. In other words, the model can be used to identify suited operating conditions, saving experimental screening time and material usage.

**Figure 9:**
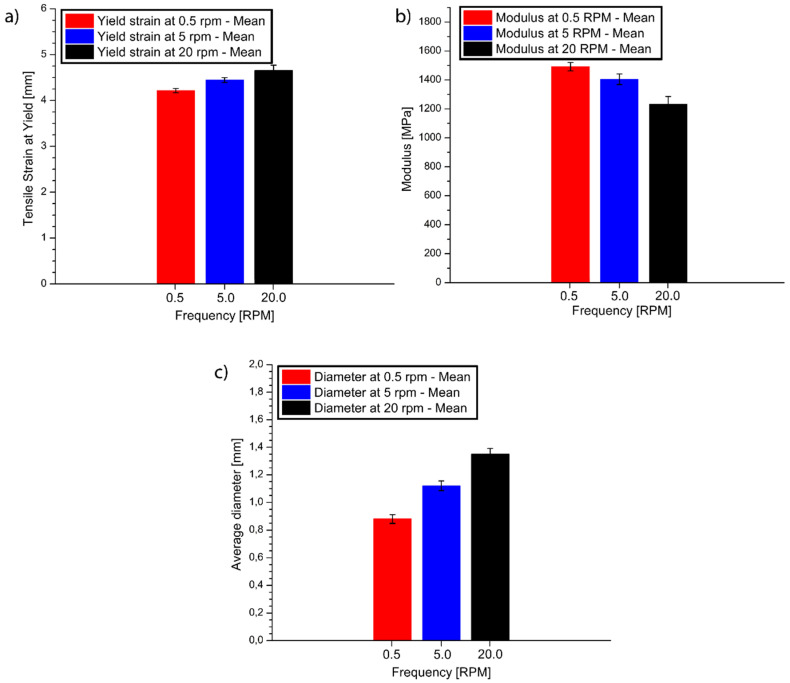
(**a**) Tensile strain; (**b**) modulus; (**c**) final diameter for ABS filament extruded at 0.5, 5, 20 rpm. The other operating conditions are given in Table 3.

Furthermore, the tensile curves in Figure S18 of the Supplementary Materials do not show substantial differences among the different frequencies. It is worthwhile mentioning that the amount of solid material predicted by the numerical model for the micro-extrusion at 20 rpm was under 1% and, due to the high temperature, the melting should have been completed in the filament after the extrusion. A slight increment in the yield strain and a decrement in the modulus with increasing frequency was observed, as shown in Figure 9a,b. These variations can be attributed to the variations in the average diameters of the filaments, caused by the different die swell, as visible in Figure 9c and coherent with previous extrusion work [64]. For higher frequencies, the larger diameters cause an increment in the cooling time, allowing the molecules to partially lose orientation, causing the drop in modulus. During the tensile test, the molecules can then be re-oriented, leading to a higher strain at yield for the larger diameters, and therefore higher frequencies.

## 5. Conclusions

Once rheological data are available, an EAM melting model, based on the drag removal principle and accounting for both conduction and heat dissipation, can be utilized to predict melting efficiencies. This has been confirmed by acceptably describing screw-freezing experiments for ABS under various screw frequencies and for two other polymeric materials, SEBS and PLA, for a fixed screw frequency. Notably, the model can wipe out natural experimental discrepancies, due to the impossibility to sometimes embed screw-freed samples. In any case, reliable model parameters on physicochemical parameters are needed, although sometimes well-measured averages suffice.

The EAM melting model can also be employed to perform the design, and here the material type plays a role. A sufficiently high processing temperature is needed to ensure sufficiently low viscosities. The model also allows us to establish the melting mechanism of the studied materials; for ABS only, a short period of a dominant contribution from heat dissipation was observed. The screw-freezing experiments further showcase that under several conditions, an outer circulation flow is present related to the geometry and dimensions of the micro-extruder.

The SEM analysis of the extruded filaments for a set of operating conditions that have a predicted point of melt finalization outside of the envisaged melting zone presents a higher amount of surface defects compared to the filaments obtained via operating conditions with a melting point well within the extruder’s limits. These defects can be attributed to the presence of unmolten material within the extruder, confirming the importance of the melting model in the process design of polymeric AM applications. Furthermore, the screw frequency also has an impact of the die swell and the tensile properties.

Hence, the current work highlights the relevance of controlling the melting in the overall process and the need for further material-oriented research to fully design the printing process.

## Figures and Tables

**Table 1:** Material characteristics for ABS (column 2), PLA (column 3), and SEBS (column 4); “melting” temperature (*T*_m_) based on DSC measurements (see Section 3.2); glass transition temperature (*T*_g_). Data from Refs. [50,51,52,53,54,55,56,57].

	ABS	PLA	SEBS	Unit
Density (at 300 K)	1.01–1.08	1.22–1.30	0.87	[g/cm^3^]
*T*_g_	381-382	326–337	183 (Butadiene)–373 (Styrene)	[K]
*T*_m_	443–593	418–459	455–553	[K]
Tensile strength, break	22–59.3	59	1.80–12.00	[MPa]
Elongation at break	2.5–40	<10%	310–860	[%]
Modulus of Elasticity	1.5–2.6	3.5	0.6–4.68	[GPa]
Morphology	Amorphous	Semicrystalline	Thermoplastic elastomer	

**Table 2:** Machine geometry and dimensions for extrusion-based additive manufacturing in the present work.

Characteristic			Unit
Axial length	*L*_a_	662	[mm]
- Feeding	*L*_f_	280	[mm]
- Compression	*L*_c_	274	[mm]
- Metering	*L*_m_	108	[mm]
Screw diameter-initial	*D*_i_	28	[mm]
Screw diameter-final	*D*_f_	16	[mm]
Helical length	*L*_h_	690	[mm]
Channel depth-initial	*H*_i_	6	[mm]
Channel depth-final	*H*_f_	2	[mm]
Channel width	*W*	16	[mm]
Pitch angle	*Θ*	0.308	[rad]

**Table 3:** Summary of materials properties and operating conditions.

**Material Properties**		**ABS**	**PLA**	**SEBS**	**Unit**	**Section**
Cross model parameter	*η*_0_	115,126	1755	10,700	[Pa s]	Section 3.1
Cross model parameter	τ*	49,521	265,954	79,318	[Pa]	Section 3.1
Cross model temperature	*T*_cr_	483	453	463	[K]	Section 3.1
Pseudo-plasticity index	*N*	0.24	0.1856	0.39	[/]	Section 3.1
Specific heat capacity solid	*c*_s_	1273	1315	1993	[J kg^−1^ K^−1^]	Section 3.2
Specific heat capacity melt	*c*_m_	2277	2425	2607	[J kg^−1^ K^−1^]	Section 3.2
Specific heat capacity (avg.)	*c*	1775	1870	2300	[J kg^−1^ K^−1^]	Section 3.2
Thermal conductivity (avg.)	K	0.191	0.227	0.331	[W m^−1^K^−1^]	
Heat of fusion	λ	0	47,000	0	[J kg^−1^]	Section 3.2
“Melt” temperature	*T*_m_	423	442	423	[K]	Section 3.2
Glass transition temperature	*T*_g_	373	331	238	[K]	Section 3.2
Density (solid)	*ρ*_s_	1050	1240	910	[kg m^−3^]	Section 3.2
Density (melt)	*ρ*_m_	979	1119	796	[kg m^−3^]	Section 3.3
**Operating Conditions**		**ABS**	**PLA**	**SEBS**	**Unit**	**Section**
Initial Temperature	*T*_0_	300	300	300	[K]	/
Barrel Temperature	*T*_b_	483	453	463	[K]	/
Screw frequencies (for model validation)	*N*	2,5,8	5	5	[rpm]	/
Volumetric inlet flow at 2 rpm	*Q*_0_	141.3	-	-	[mm^3^ s^−1^]	/
Volumetric inlet flow at 5 rpm	*Q*_0_	104.3	98.1	109.4	[mm^3^ s^−1^]	/
Volumetric inlet flow at 8 rpm	*Q*_0_	47.1	-	-	[mm^3^ s^−1^]	/

**Table 4:** Thermal conductivity measurements for ABS, PLA, and SEBS from Hot disk tests.

Material ^1^	Temperature [K]	Measured *k* [W m^−1^ K^−1^]	Error [W m^−1^ K^−1^]
ABS	296	0.181	±0.0003
ABS	373	0.200	±0.0010
PLA	296	0.224	±0.0020
PLA	383	0.229	±0.0030
SEBS	296	0.331	±0.001

^1^ The experiments were conducted according to ASTM standard E1269 at 50% relative humidity.

**Table 5:** Specific heat capacity for ABS, PLA, and SEBS from DSC at a heating rate of 5 K min^−1^.

		Temperature [K]	ABS	PLA	SEBS	Unit
Specific heat capacity	*c*_s_	300	1273	1315	1993	[J kg^−1^ K^−1^]
Specific heat capacity	*c*_m_	460	2277	2425	2607	[J kg^−1^ K^−1^]

**Table 6:** Measurements toward melt density for ABS, PLA, and SEBS.

Material	MVR [cm^3^/10 min]	Error [cm^3^/10 min]	Extruded Weight [g/10 min]	Temperature [K]	External Weight [kg]	Melt Density [g/cm^3^]
ABS	8.187	±0.401	7.910	493	10	0.966
ABS	8.809	±0.138	8.727	493	10	0.991
PLA	6.975	±0.150	7.852	473	2.16	1.126
PLA	7.365	±0.286	8.186	473	2.16	1.111
SEBS	3.701	±0.027	2.930	473	2.16	0.792
SEBS	3.639	±0.032	2.908	473	2.16	0.799

## Data Availability

Not applicable.

## Supplementary Materials

The following are available online at https://www.mdpi.com/article/10.3390/ma14195566/s1, Figure S1: Definition of x and z direction for the general equations for melt removal by drag, Figure S2: DSC for the PLA pellets and PLA injection molded hot disk sample. The melting temperature was defined as the onset temperature of the melting peak for pellets, Figure S3: Examples of sections of the solidified extruder channel in the micro extruder for a) PLA; b) SEBS, Figure S4: Image processing for ABS 5RPM (not embedded sample 1), Figure S5: Image processing for ABS 5RPM (not embedded sample 2), Figure S6: Image processing for ABS 5 RPM (embedded sample), Figure S7: Image processing for ABS 2RPM (embedded sample), Figure S8: Image processing for ABS 8RPM (embedded sample), Figure S9: Image processing for PLA 5 RPM (not embedded sample), Figure S10: Image processing for SEBS 5RPM (not embedded sample), Figure S11: (a) Relevance of the Q0 parameter; (b) Relevance of the n parameter, Figure S12: SEM Images for ABS extruded filament at 0.5 rpm, 0.8 mm nozzle, Figure S13: SEM Images for ABS extruded filament at 5 rpm, 0.8 mm nozzle, Figure S14: SEM Images for ABS extruded filament at 20 rpm, 0.8 mm nozzle, Figure S15: SEM Images for ABS at 0.5 rpm, 1.5 mm nozzle, Figure S16: SEM Images for ABS at 5 rpm, 1.5 mm nozzle, Figure S17: SEM Images for ABS at 20 rpm, 1.5 mm nozzle, Figure S18: Tensile curves for ABS filament extruded at a) 0.5 rpm; b) 5 rpm; c)20 rpm. Other operating conditions in Table 3 of the main document.
