# Supplementary material for: A Combined Experimental and Modeling Study for Pellet-Fed Extrusion-Based Additive Manufacturing to Evaluate the Impact of the Melting Efficiency
Source: Materials (Basel). 2021 Sep 25;14(19):5566. doi: 10.3390/ma14195566 (PMC8509700; doi:10.3390/ma14195566)
Supplement: Supplementary file 1 [file materials-14-05566-s001.zip › materials-1366580-supplementary.pdf]

materials

Supplementary Information for 'A Combined Experimental and Modeling Study for Pellet-Fed Extrusion-Based Additive Manufacturing to Evaluate the Impact of the Melting Efficiency'

Andrea La Gala [1], Rudinei Fiorio [1], Daniel V. A. Ceretti [1], Mustafa Erkoç [1], Ludwig Cardon [1] and Dagmar R. D'hooge [2,3,*]

[1] Centre for Polymer and Material Technologies (CPMT), Department of Materials, Textiles and Chemical Engineering, Ghent University, Technologiepark, 130, Zwijnaarde 9052, Ghent 9000, Belgium; Andrea.LaGala@UGent.be (A.L.G.); rudinei.fiorio@UGent.be (R.F.); daniel.ceretti@ugent.be (D.V.A.C.); mustafa.erkoc@ugent.be (M.E.); ludwig.cardon@ugent.be (L.C.)
[2] Centre for Textiles Science and Engineering (CTSE), Department of Materials, Textiles and Chemical Engineering, Ghent University, Technologiepark, 70A, Zwijnaarde 9052, Ghent 9000, Belgium
[3] Laboratory for Chemical Technology (LCT), Department of Materials, Textiles and Chemical Engineering, Ghent University, Technologiepark, 125, Zwijnaarde 9052, Ghent 9000, Belgium
* Correspondence: dagmar.dhooge@ugent.be

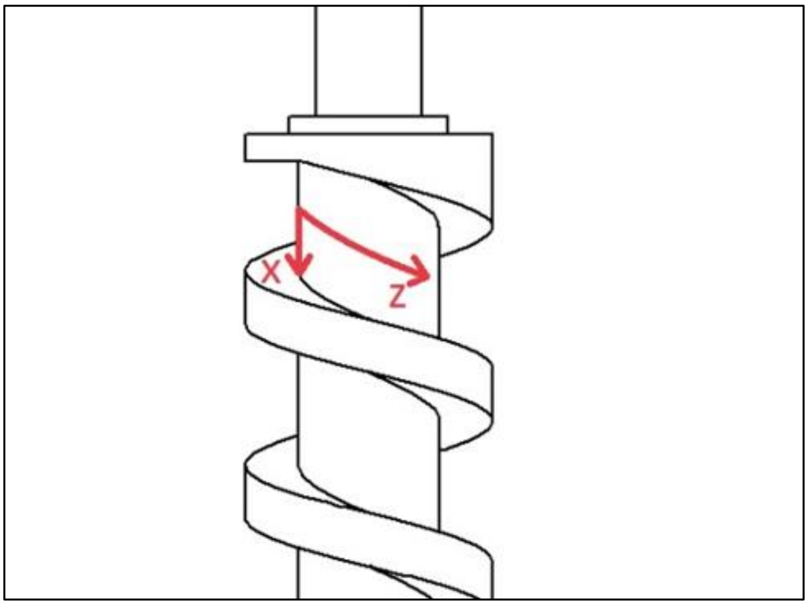

Figure S1 - Definition of x and z direction for the general equations for melt removal by drag.

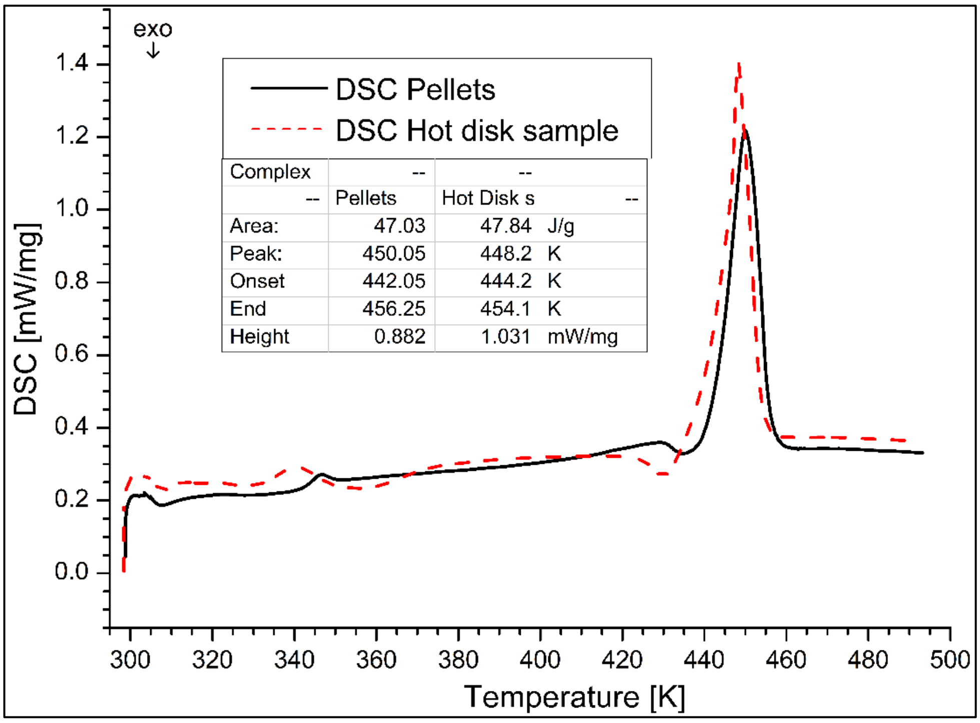

Figure S2 - DSC for the PLA pellets and PLA injection molded hot disk sample. The melting temperature was defined as the onset temperature of the melting peak for pellets.

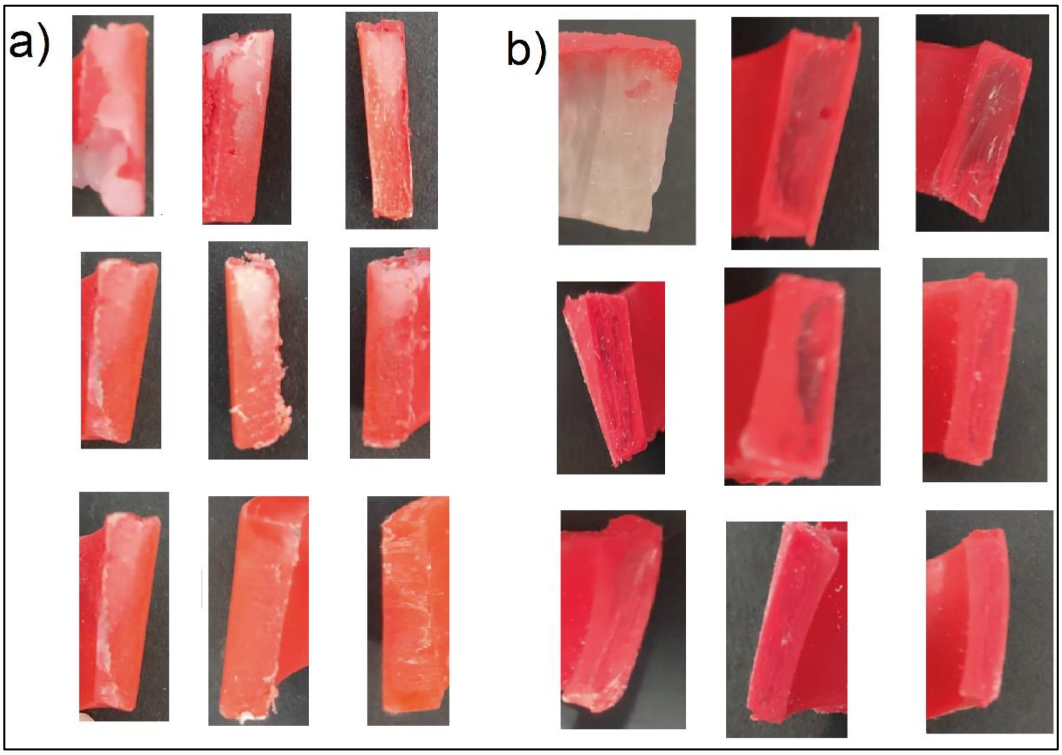

Figure S3- Examples of sections of the solidified extruder channel in the micro extruder for a) PLA; b) SEBS.

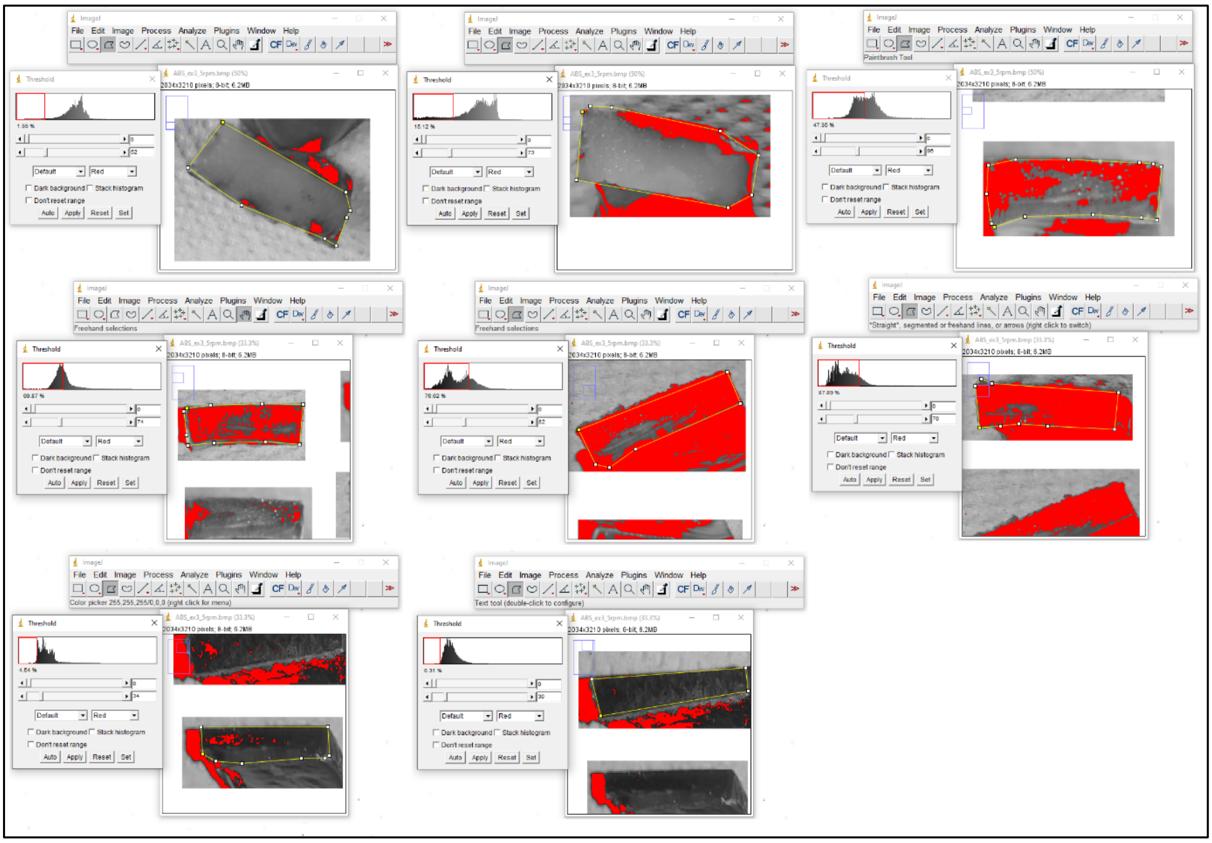

Figure S4 - Image processing for ABS 5RPM (not embedded sample 1).

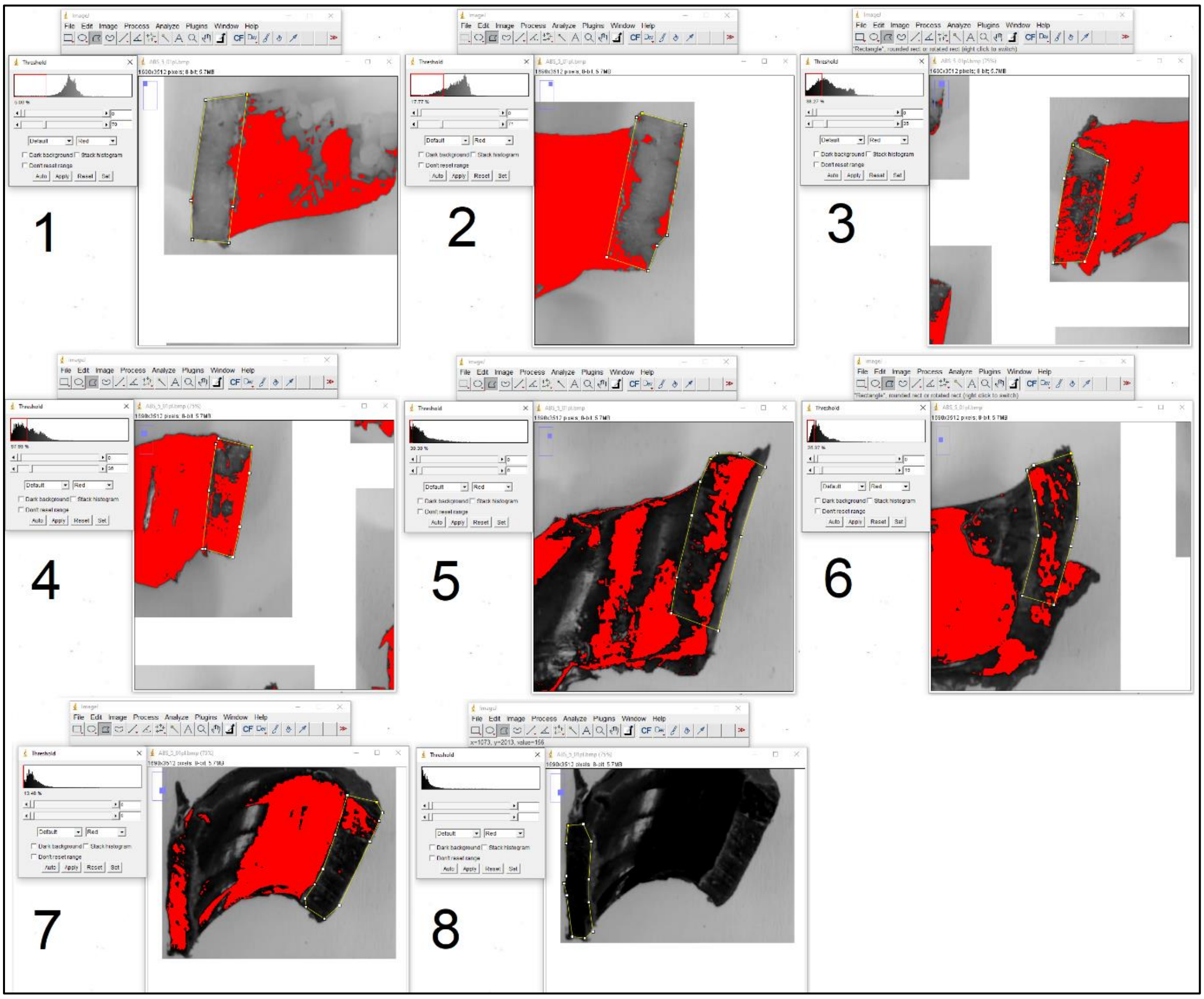

Figure S5 - Image processing for ABS 5RPM (not embedded sample 2).

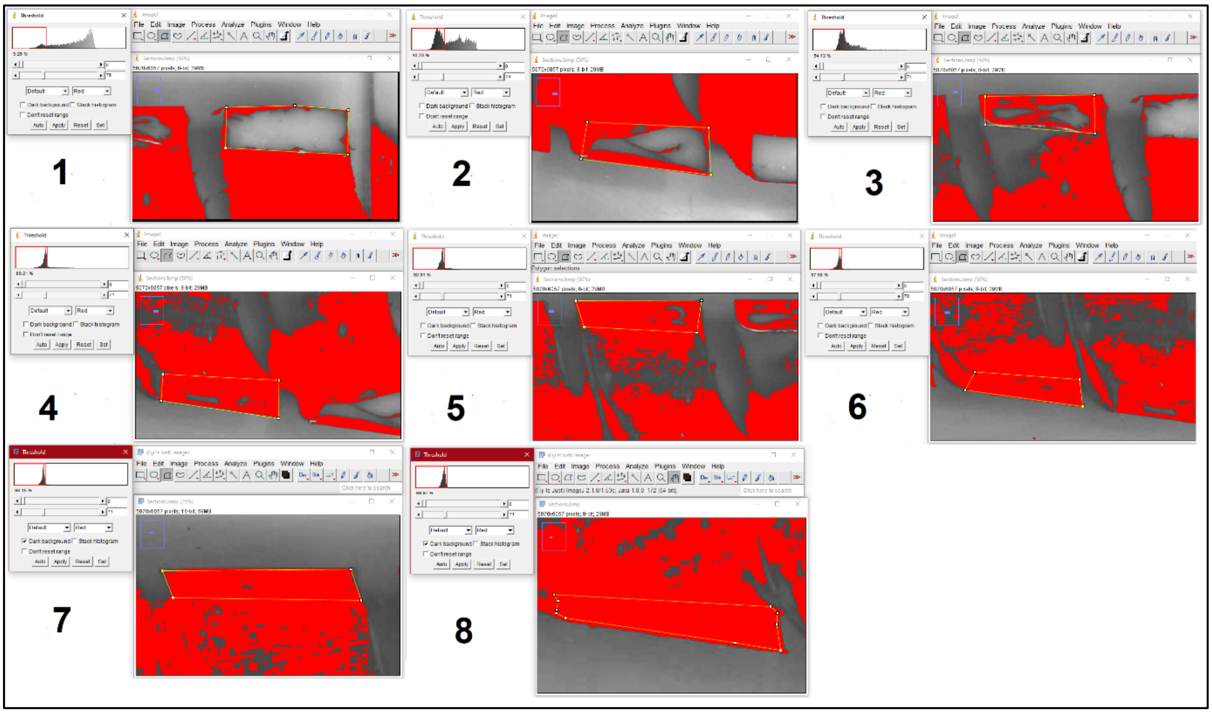

Figure S6- Image processing for ABS 5 RPM (embedded sample).

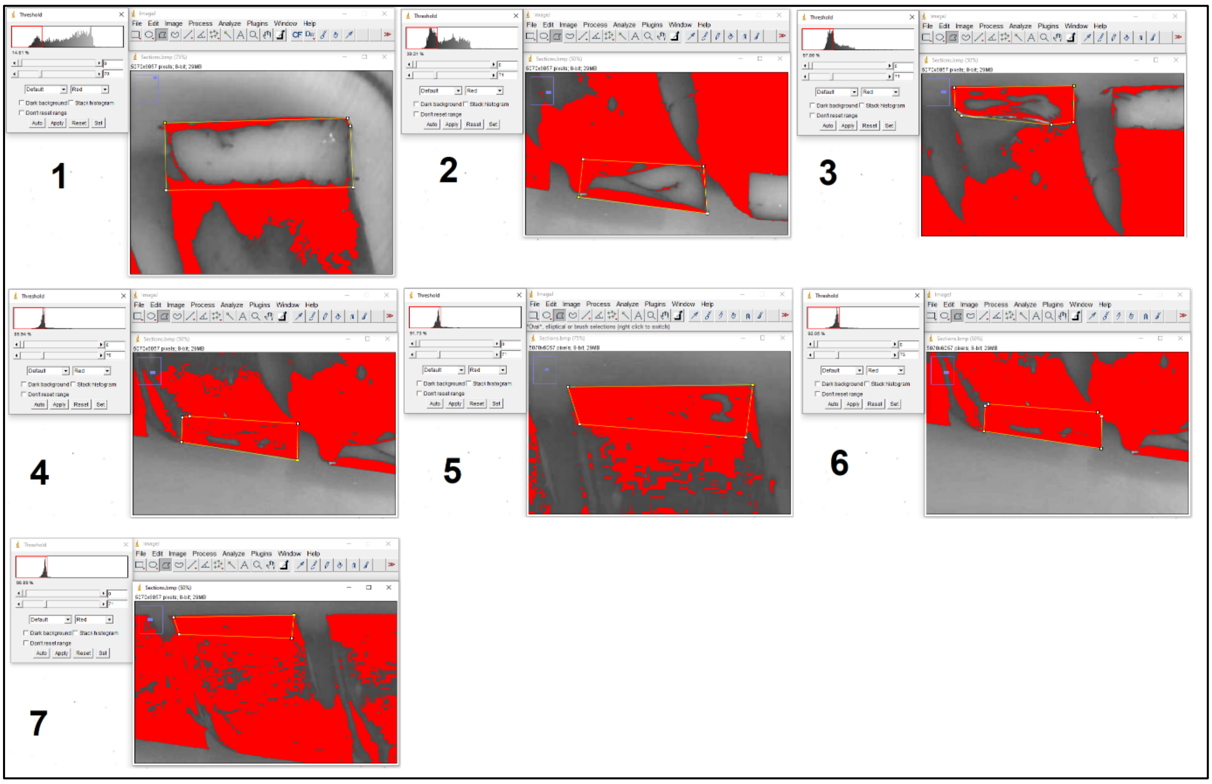

Figure S7 - Image processing for ABS 2RPM (embedded sample).

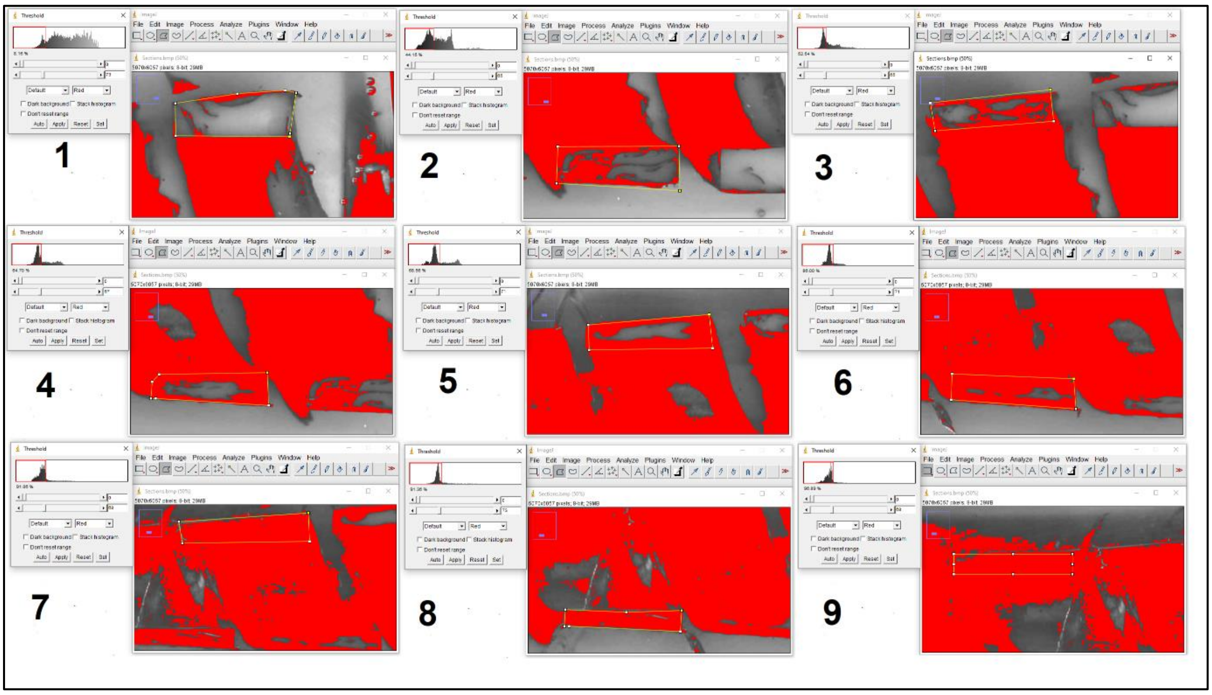

Figure S8- Image processing for ABS 8RPM (embedded sample).

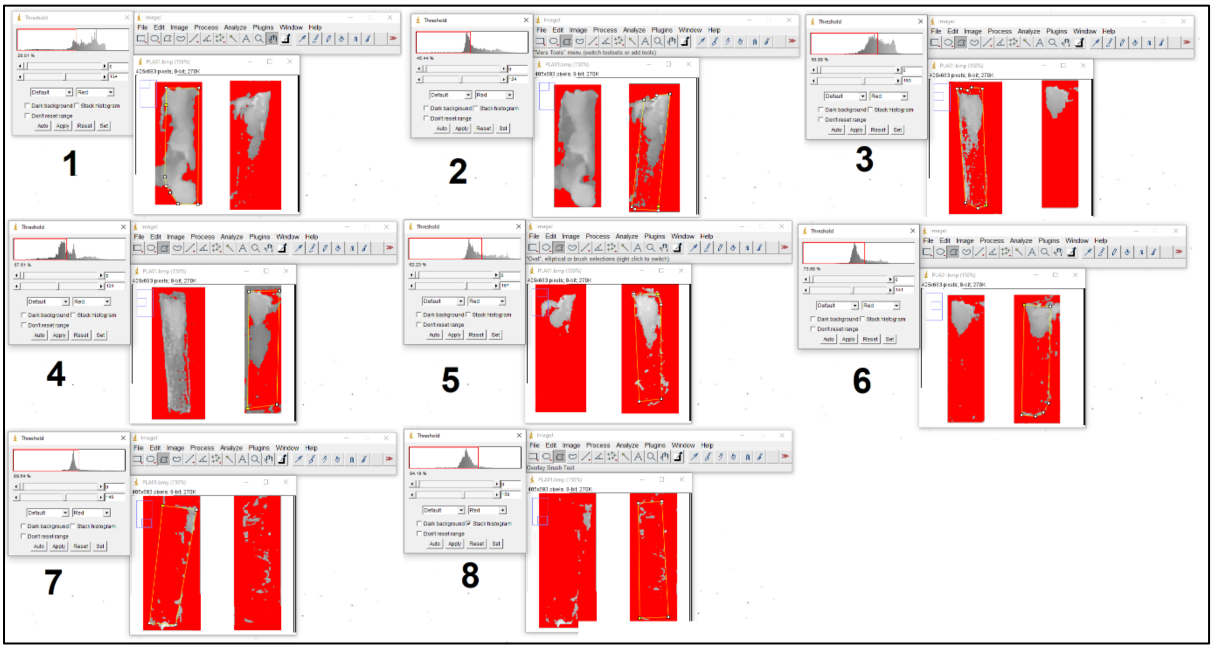

Figure S9- Image processing for PLA 5 RPM (not embedded sample).

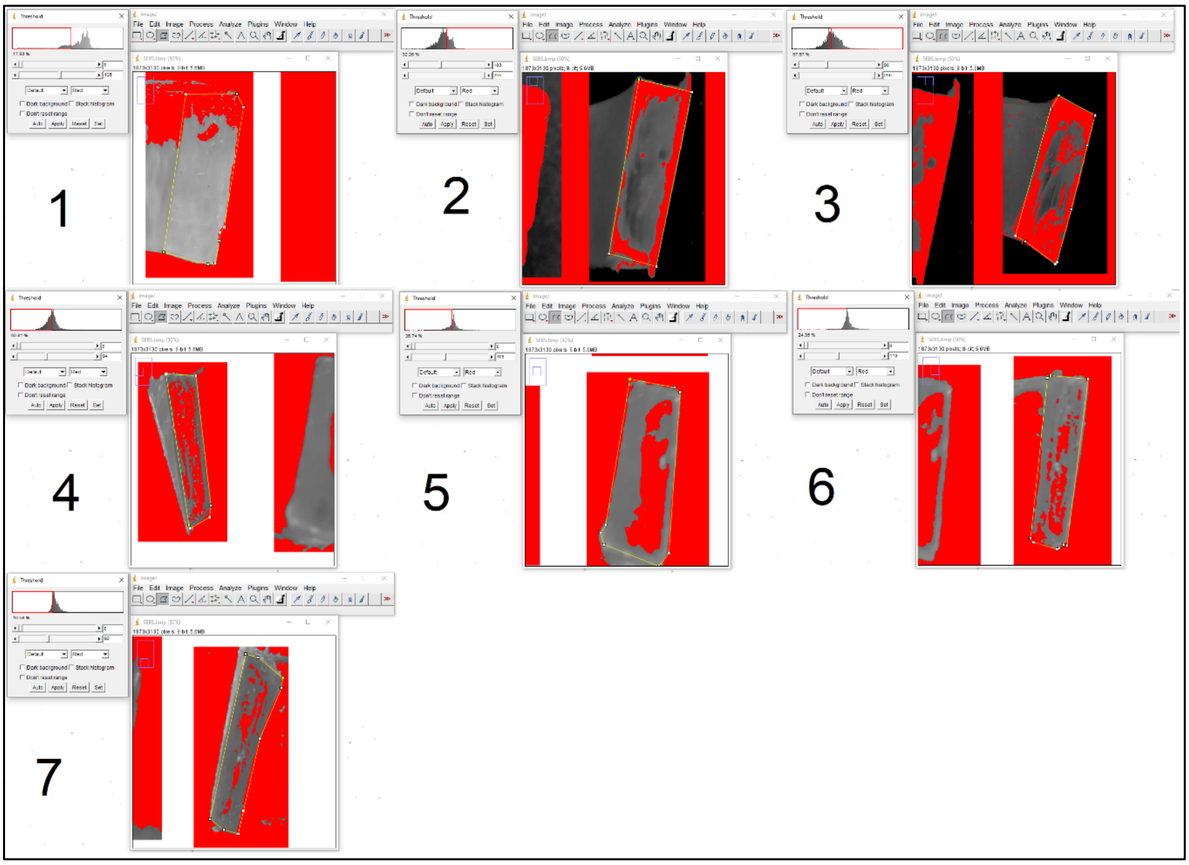

Figure S10- Image processing for SEBS 5RPM (not embedded sample).

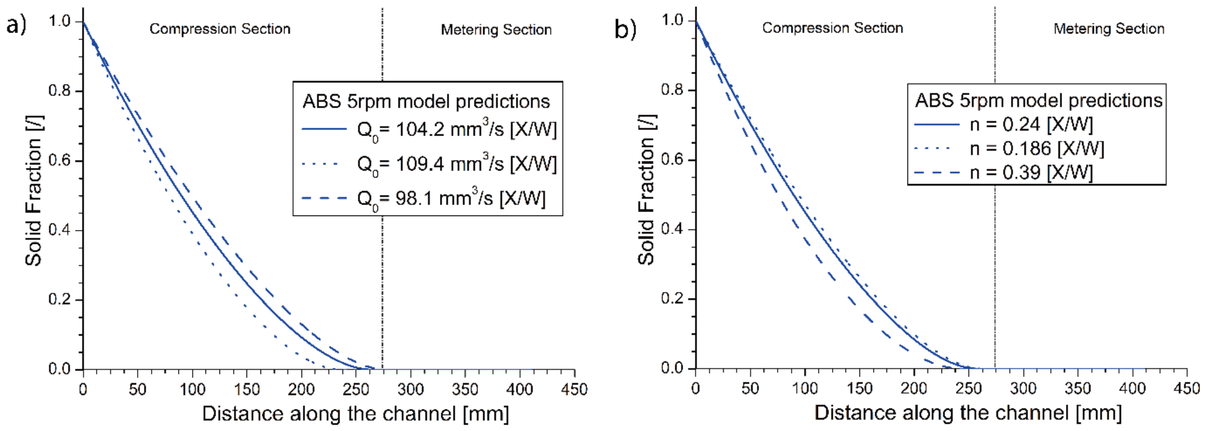

Figure S11 – (a) Relevance of the Q_0 parameter; (b) Relevance of the n parameter.

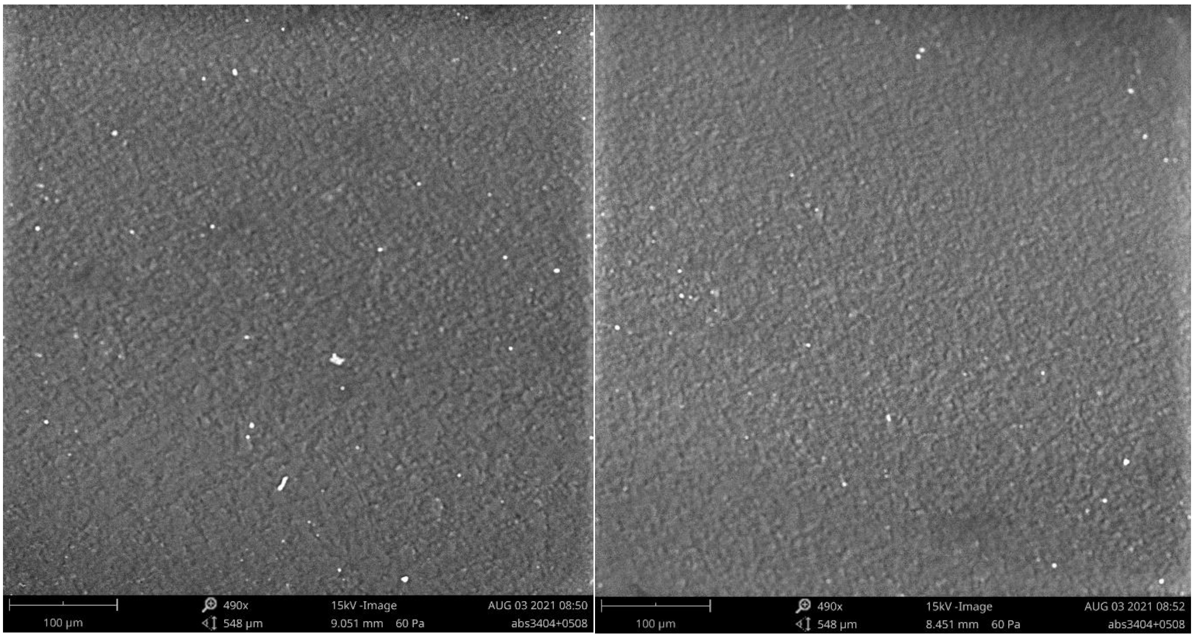

Figure S12- SEM Images for ABS extruded filament at 0.5 rpm, 0.8mm nozzle.

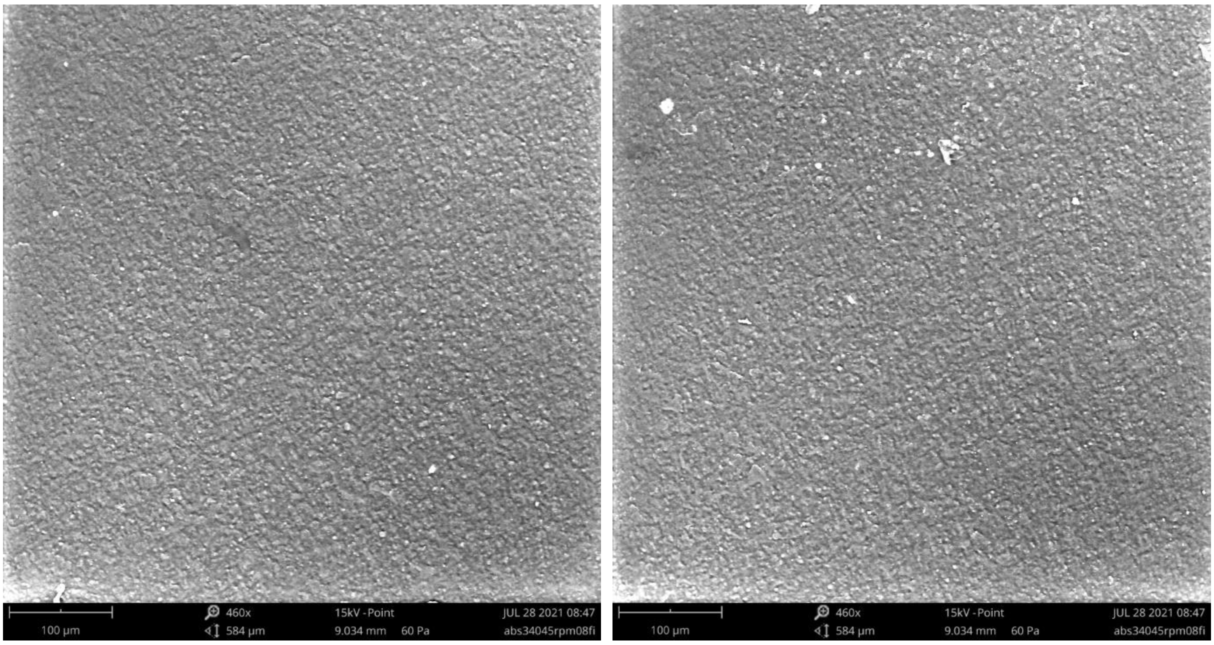

Figure S13 - SEM Images for ABS extruded filament at 5 rpm, 0.8mm nozzle.

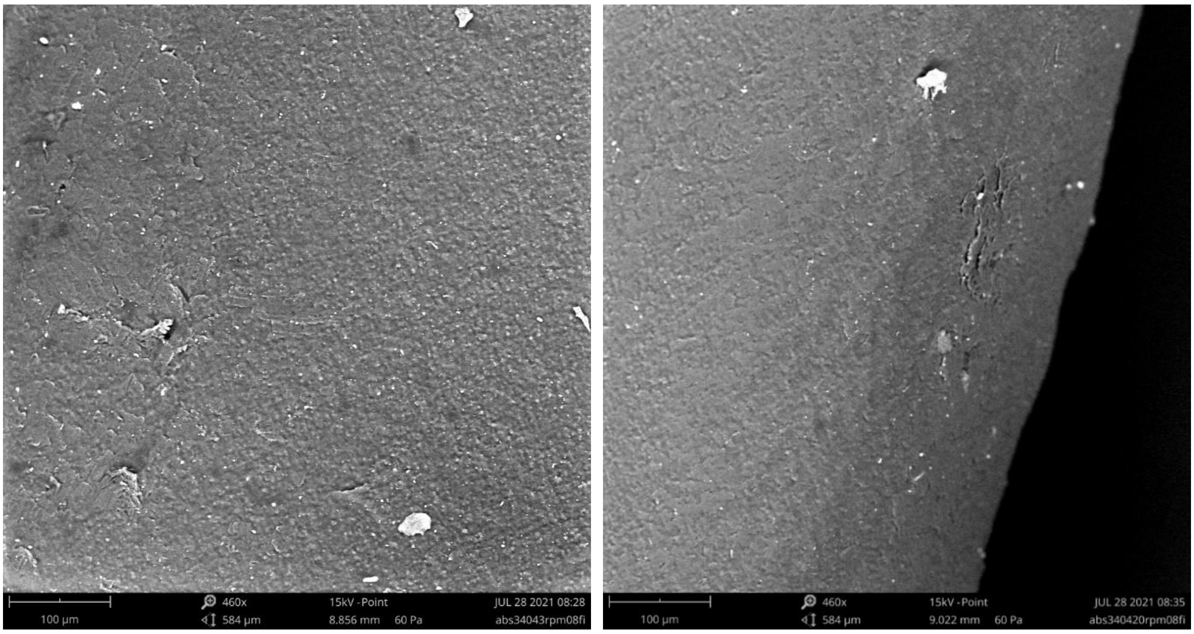

Figure S14- SEM Images for ABS extruded filament at 20 rpm, 0.8mm nozzle.

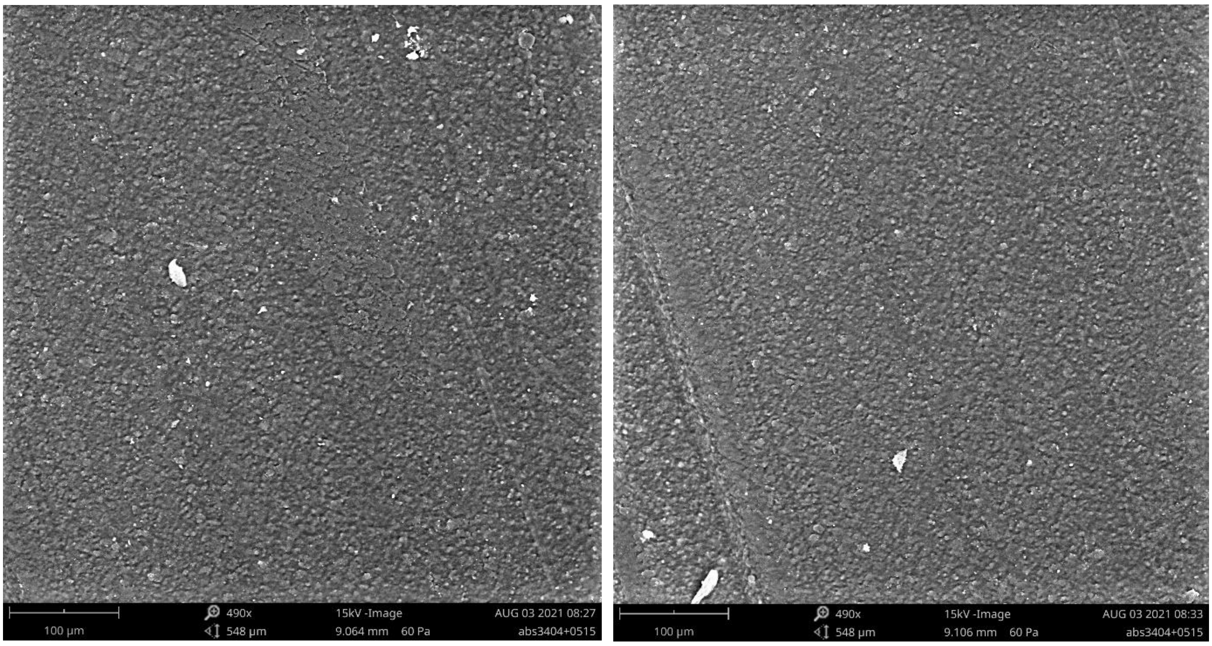

Figure S15 - SEM Images for ABS at 0.5 rpm, 1.5mm nozzle.

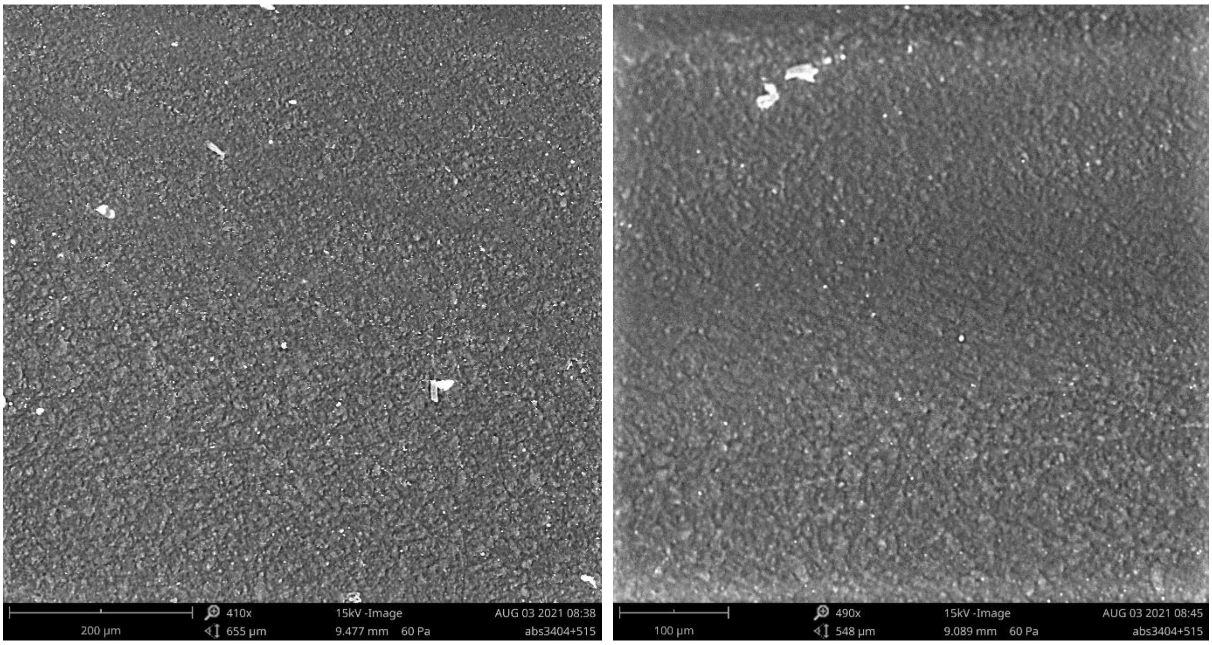

Figure S16 - SEM Images for ABS at 5 rpm, 1.5mm nozzle.

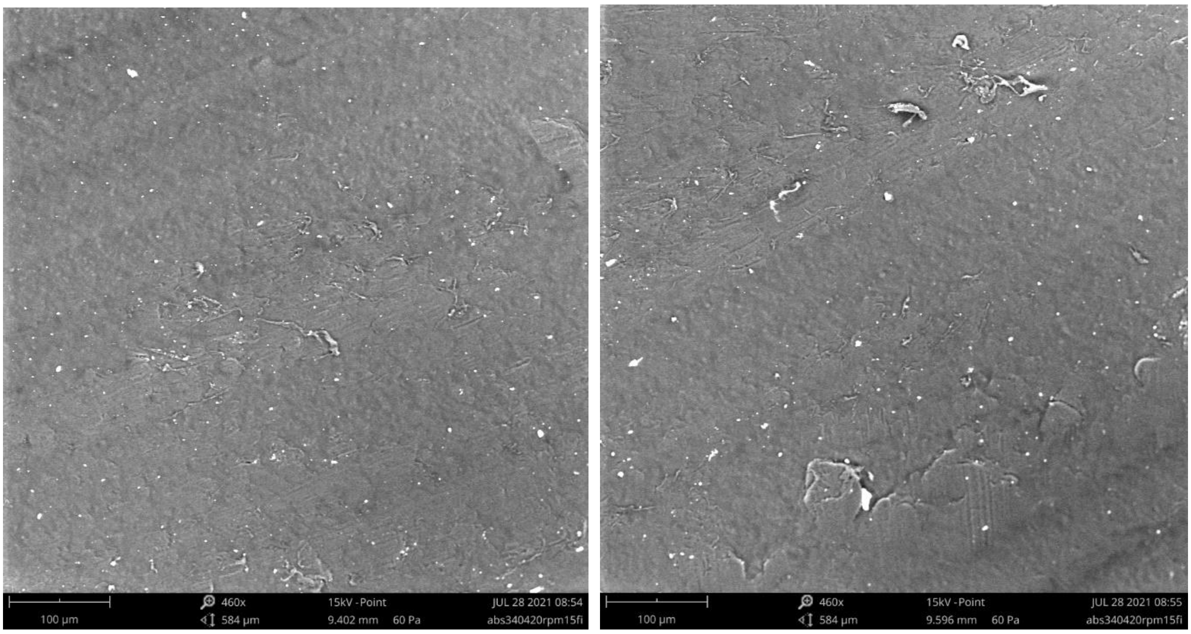

Figure S17 - SEM Images for ABS at 20 rpm, 1.5mm nozzle.

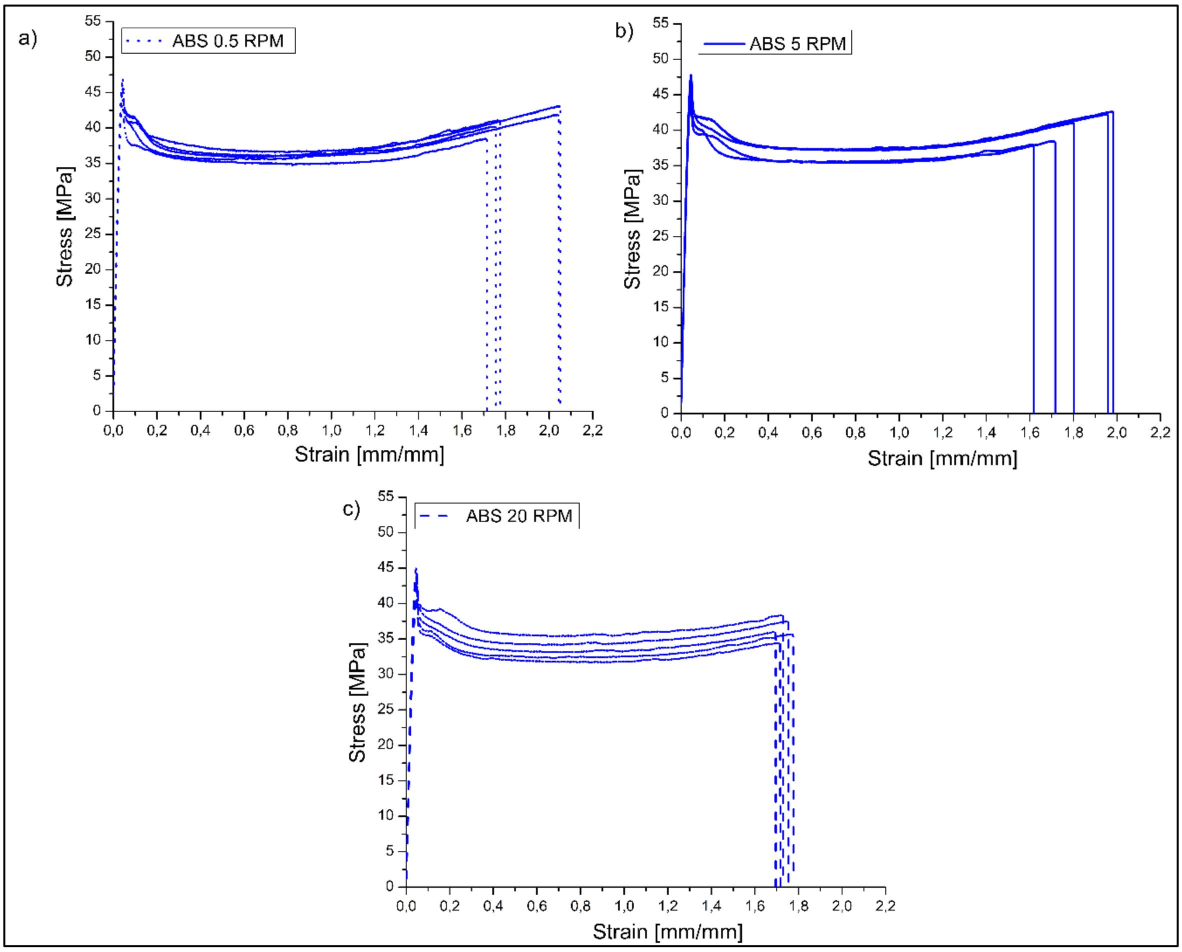

Figure S18 Tensile curves for ABS filament extruded at a) 0.5 rpm; b) 5 rpm; c)20 rpm. Other operating conditions in Table 3 of the main document.
